# Assessment of Blood Glucose Measurement Using New Noninvasive Technology: Protocol and Methodology

**DOI:** 10.2196/76558

**Published:** 2026-01-08

**Authors:** Eka Widrian Suradji, Satvinder Singh Dhaliwal, Zhang Li-Feng, Entong Zhou, Amos Lim, Hui Hern Wong, Yao Du, Fredicia Fredicia, Amanda Dianky, Puspasari Pasaribu, Ernest Gamaliel Masayoshi Takashi, Justin Yong An Tan, Mardi Santoso, Seng Bin Ang

**Affiliations:** 1 Department of Public Health, Faculty of Medicine and Health Sciences, Krida Wacana Christian University, Arjuna Utara no 6, Kec Kebon Jeruk, West Jakarta, DKI Jakarta, 11510, Indonesia, 62 (021) 56942061; 2 Integrated Research Laboratory, Krida Wacana Christian University, DKI Jakarta, Indonesia; 3 LIF Indonesia, DKI Jakarta, Indonesia; 4 Curtin Medical Research Institute, Curtin University, Curtin, Australia; 5 Obstetrics and Gynaecology Academic Clinical Program, Duke-NUS Medical School, Singapore, Singapore; 6 Office of the Provost, Singapore University of Social Sciences, Singapore, Singapore; 7 Health and Social Sciences, Singapore Institute of Technology, Singapore, Singapore; 8 Actxa Pte Ltd, Singapore, Singapore; 9 Computer Science Department, Faculty of Engineering and Computer Science, Krida Wacana Christian University, DKI Jakarta, Indonesia; 10 Applied Artificial Intelligence, School of Science, Singapore Institute of Technology, Singapore, Singapore; 11 Department of Internal Medicine, Faculty of Medicine and Health Sciences, Krida Wacana Christian University, DKI Jakarta, Indonesia; 12 Family Medicine Academic Clinical Program, Duke-NUS Medical School, Singapore, Singapore; 13 Family Medicine, KK Women's and Children's Hospital, Singapore, Singapore

**Keywords:** diabetes mellitus, noninvasive, blood glucose, screening, machine learning, photoplethysmography, artificial intelligence, wearables, BGEM, Indonesia, Blood Glucose Evaluation and Monitoring

## Abstract

**Background:**

Diabetes mellitus (DM) is a major noncommunicable disease with a significant increase in prevalence, especially in low- and middle-income countries. The latest International Diabetes Federation Diabetes Atlas (2025) reports that 11.1% of the adult population (20 to 79 years old) is living with diabetes, with over 4 in 10 unaware of their condition. Early diagnosis and treatment of diabetes reduce the risk and slow the progression of debilitating complications, such as amputation, vision loss, renal failure, cardiovascular disease, dementia, some cancers, and infections like tuberculosis and severe COVID-19. Current screening methods for diabetes are invasive and costly. This has limited their utilization, especially in high-density populations and low- and middle-income countries such as Indonesia. Blood Glucose Evaluation and Monitoring (BGEM) is a machine learning algorithm developed by Actxa to analyze photoplethysmography data from wearable devices for diabetic risk assessment. Its noninvasive and user-friendly nature makes it a strong candidate for fulfilling the need for a diabetes screening or monitoring tool.

**Objective:**

The aim of this study is to collect a large and more diverse dataset for the training of BGEM machine learning models. This dataset is intended to improve the model’s generalizability and to evaluate its performance across different age groups, racial groups, and skin types, with the goal of enhancing accuracy and robustness for diabetes risk assessment and glucose monitoring.

**Methods:**

Adult participants aged 18 years and above, with either a diabetic or a nondiabetic history, who reside in Greater Jakarta Area, Indonesia, were approached for recruitment. Blood glucose was assessed using laboratory blood analysis from capillary or plasma samples after fasting and at 1, 2, and 3 hours after a meal. BGEM data were also collected at each of these time points. Anthropological measurements with a standardized questionnaire on physical activity, demographic information, respondent’s diabetic status, and current medications taken were also collected.

**Results:**

Between June and October 2024, 885 participants were enrolled. Eight photoplethysmography recordings per participant were collected across 4 meal time points using 2 wearable devices in addition to the collection of clinical measurements, blood sampling, and related questionnaires.

**Conclusions:**

This protocol paper outlines the methodology designed for assessing and interpreting participants’ blood sugar profiles, especially on demographic variability, in order to evaluate BGEM, a photoplethysmography-based artificial intelligence model designed to estimate blood glucose levels and diabetic risk. The clinical trial was conducted on Indonesian participants with and without diabetes while considering various influencing factors. This dataset is designed to enable assessment of the model’s performance across diverse racial, risk factors, and skin-type groups, with the aim of making the model more valid and reliable.

## Introduction

Diabetes mellitus (DM) is a major noncommunicable disease that has affected around 500 million people worldwide and continues to rise up to 700 million people globally in 2045 [1]. Other noncommunicable diseases’ death risks have decreased by 20% since the turn of the century; DM, in contrast, has increased by 3% worldwide. The latest International Diabetes Federation Diabetes Atlas (2025) reports that 11.1% of the adult population (20‐79 y) is living with diabetes, with over 4 in 10 unaware of their condition.

Several reports have projected that about 1 in 2 adult patients with diabetes are unaware of their diagnosis [1-3]. Undiagnosed DM has resulted in an increase of cases with complications and elevated the burden of the disease, as younger undiagnosed DM cases have more rapid progression of complications and lower response to general DM treatment. This has been shown to be higher in low- and middle-income countries, where 80% of the DM population lived, thus reflecting the enormous burden of the disease [4,5]. Meanwhile, early diagnosis and treatment of DM reduce the risk and slow the progression of debilitating complications such as amputation, vision loss, renal failure, cardiovascular disease, dementia, some cancers, and infections including tuberculosis and severe COVID-19 [6]. All of the above calls for early screening and frequent monitoring strategies that are both effective and cost-efficient.

Currently available methods to detect DM are hemoglobin A_1c_ (HbA_1c_) level, fasting plasma glucose, 2-hour glucose tolerance test, and random plasma glucose test. All these methods are invasive; they require trained professionals and laboratory equipment to be performed correctly. The American Diabetes Association has recommended DM screening starting at 35 years and every 3 years after, especially for people with obesity, overweight, and one risk factor [7]. In low- and middle-income countries, this is even harder to implement due to the cost and scarce resources of such screening methods [5,6,8].

Indonesia, a middle-income country with a population of 283 million, faces a big challenge in DM management. It was estimated that DM prevalence was 9.49% (2024) and will increase to 16.09% (2045) without any major effective intervention [9]. As in any other low- and middle-income countries, the cost and availability of DM-related testing have prohibited Indonesia from enacting an effective prevention or screening program; therefore, an affordable, effective, and simple screening or monitoring method for DM will be a boon in the national health program.

Photoplethysmography is a noninvasive optical technology that measures light absorbance to assess blood volume changes. It has been widely integrated into wearable devices, such as smartwatches, to monitor heart rate (HR) and other physiological parameters [10]. Emerging research suggests that photoplethysmography data may also be valuable for detecting DM and estimating glucose levels [11-13]. The noninvasive nature, affordability, and convenience of photoplethysmography make it a promising tool for early detection and preventive screening of DM, especially in large and diverse populations, such as in Indonesia. Despite photoplethysmography technology’s potential use in estimating blood glucose and DM prediction, several issues have been reported to influence its performance, such as medication status of subjects [14], motion artifacts [15], and also skin types [16]. This protocol paper will strive to address these issues to improve the reliability (accuracy and robustness) of photoplethysmography-based technology in wearable devices for blood glucose monitoring and DM risk prediction through advanced artificial intelligence (AI) and data analytics.

Blood Glucose Evaluation and Monitoring (BGEM), a machine learning (ML) algorithm developed by Actxa to analyze photoplethysmography data for assessing diabetic risk. It is one of the first ML models deployed in commercial wearable devices. BGEM was trained using data collected from a registered clinical trial (NCT05504096) conducted at KK Women’s and Children’s Hospital in Singapore, involving 500 Singaporean participants. The reported accuracy of BGEM is 84.7% [12]. The model’s performance is sensitive to demographic features, with the inclusion of demographic data significantly enhancing its accuracy [13]. BGEM would benefit from a larger and more diverse training dataset, as the current training data consists primarily of Singaporean participants, predominantly healthy females.

In addition to glycemia prediction, novel methods for diabetic risk assessment and management based on multiple glucose measurements may be developed using BGEM due to its lower cost and noninvasiveness. Glycemic variability, which refers to fluctuations of blood glucose concentration that happen in a day or similar timeframes on different days, has been shown to be associated with microvascular and macrovascular damage in patients with DM [17]. Glycemic variability has been proposed in multiple studies for DM management; however, difficulties in the measurement and consensus on its optimal value, even with recent more widespread use of continuous glucose monitoring, have limited its use in the general population [17,18], despite the general acceptance that it is a promising indicator for DM control.

Here, we present the protocol for a registered clinical trial (NCT06642467) designed to collect a larger and more robust training dataset to enhance BGEM’s performance. This study will collect data to improve current algorithm performance, with an attempt to generalize the diagnostic findings from the previous clinical study to specific groups of participants, with the goal of enhancing accuracy and robustness for glucose monitoring and diabetes risk assessment. The study aims to comprehensively collect photoplethysmography data using 2 different types of wearable devices at multiple time points before and after a breakfast meal. Both capillary and plasma blood samples were collected and subjected to thorough laboratory analysis. Participants from Indonesia were recruited, ensuring a balanced representation in terms of diabetic status (diabetic or nondiabetic) and gender. Additionally, a comprehensive questionnaire was used to gather essential information, capitalizing on the diversity of the training data, including factors such as hypertension, skin type, and other relevant variables, which have been reported to possibly influence either photoplethysmography sensor readings or ML model prediction.

## Methods

### Participant Recruitment

A total number of 885 participants from adults aged 18 years and above were recruited. These participants reside in the Greater Jakarta Area, Indonesia, and were from the diabetic exercise group, from staff and family members of Krida Wacana Christian University (Ukrida), and from its affiliated organizations. Participants were recruited using an online registration provided by the research team. Exclusion criteria were implemented as follows: BMI >37 kg/m^2^, diastolic blood pressure ≥120 mmHg, and systolic blood pressure ≥180 mmHg, wearing a pacemaker, having fever or any other acute critical illness during the data collection, and pregnant female participants. All subjects received an explanation on the study purpose, protocol, and risks, and also signed the informed consent prior to admission to the research study.

### Study Design

This study utilizes a cross-sectional design with a short-term follow-up post meal for blood glucose monitoring, from July 30 to October 5, 2024, at the Ukrida Hospital, Jakarta, Indonesia. There was an a priori aim to assess the measurement error of the 4 groups, males and females with diabetes and without diabetes, separately. A sample size of 200 for each group was determined using the methods described by Bland and Altman [19]. Hence, a total sample size of 800 was required to provide a 95% CI of ±0.24×SD. Allowing for about 10% dropout, a total sample size of about 890 subjects was planned for recruitment.

**Figure 1.**
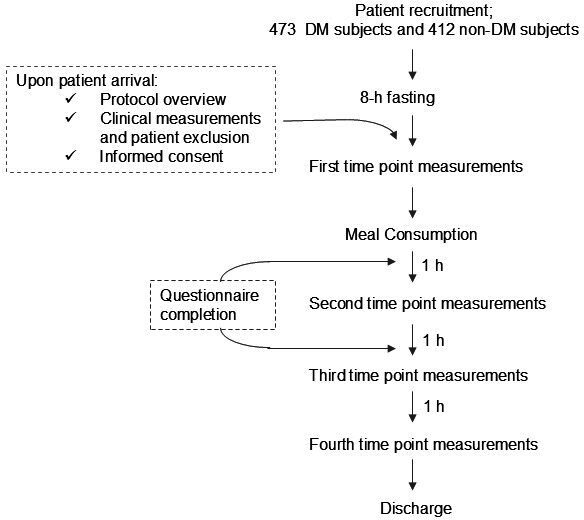
A conceptual framework of study design from recruitment, data collection, to discharge for 473 participants who self-acknowledged having diabetes mellitus (DM) and 412 participants who did not (non-DM).

The study will explore BGEM blood glucose prediction capabilities compared to laboratory blood analysis from capillary or plasma blood samples, with the study workflow summarized in Figure 1. The subjects were given an explanation on the study, asked for informed consent, and given a numeric identity tag to ensure anonymity of the dataset after undergoing a minimum of 8 hours of fasting before admittance to the study. Subjects undertook weight, height, waist circumference, and blood pressure measurement using GEA ZT120 body scales, tape measure roll, and Beurer BM26 sphygmomanometer, respectively. BMI scores were calculated from the weight and height of the subjects to evaluate obesity or metabolic condition of the subjects.

Subsequently, each participant’s photoplethysmography data at the fasting time point (the first time point) were collected using both a smart ring (JC Smart Health Ring 2301B) and a smartwatch (Actxa Spark+Series 2), both provided by Actxa Pte Ltd. Simultaneously, blood samples were obtained from 2 sources: capillary blood for glucose concentration measurement using Accu-Chek (Roche) and plasma blood from the cubital vein for laboratory analysis, as described in a later section.

Participants then consumed a 552-kcal meal set with 79.8 g of carbohydrate content prepared by Ukrida Hospital. This meal was chosen to ensure adequate sustenance and satiety after overnight fasting, as sugary drinks alone may not sufficiently fulfill. The nutritional composition of the meal is detailed in Textbox 1 and Table S1 in Multimedia Appendix 1.

Textbox 1. **Nutritional value of the set meal for the subjects, given after subjects fast for a minimum of 8 h and undergo first blood sample collection and photoplethysmography reading from wearables.**

Energy (kcal): 552.1

Water (g): 241.8

Protein (g): 19

Protein percentage: 13.8

Fat (g): 19.3

Fat percentage: 31.5

Carbohydrate (g): 79.8

Carbohydrate percentage: 57.8

Dietary fiber (g): 5.2

Polyunsaturated fatty acid (g): 1.8

Cholesterol (mg): 33

Vitamin A (µg): 1396.8

Carotene (mg): 0.4

Vitamin E (eq.) (mg): 0.8

Vitamin B1 (mg): 0

Vitamin B2 (mg): 0.1

Vitamin B6 (mg): 0.6

Total folic acid (µg): 31.8

Vitamin C (mg): 10.7

Sodium (mg): 1015.5

Potassium (mg): 549.4

Calcium (mg): 59.2

Magnesium (mg): 66.2

Phosphorus (mg): 231.5

Iron (mg): 2.5

Zinc (mg): 1.6

Participants then underwent measurements at the second time point (1 h postprandial), third time point (2 h postprandial), and fourth time point (3 h postprandial). Photoplethysmography data collection followed the same procedure as the first time point using both the smartphone and the smart ring. For blood sample collection and testing, the third time point followed the same procedure as the first time point, with both capillary and plasma blood samples collected and analyzed. At the second and fourth time points, plasma blood collection was omitted, and only capillary blood samples were obtained and tested. Further details are provided in Table 1.

**Table 1.** Blood glucose metabolism-related data collection from photoplethysmography sensors from 2 wearables, a glucometer, and blood samples based on several time points before and after meal.

Measurements	Smart ring^a^	Smartwatch^b^	Glucose meter^c^	Plasma blood^d^
First time point (fasting state)	Yes	Yes	Yes	Yes
Second time point (1 h postprandial)	Yes	Yes	Yes	No
Third time point (2 h postprandial)	Yes	Yes	Yes	Yes
Fourth time point (3 h postprandial)	Yes	Yes	Yes	No

a JC smart health ring 2301B.

b Actxa Spark + Series 2.

c Roche, ACCU-CHEK guide.

d Full blood count: Sysmex XN-550. Plasma glucose: Roche, Cobas c111 Analyzer. Hemoglobin A_1c_ level: Finecare FIA Meter III Plus.

During the 1-hour intervals at the last 3 time points, while waiting between data and sample collection, participants were asked to complete the International Physical Activity Questionnaire long form, along with questionnaires related to DM and other factors that may affect photoplethysmography reading and BGEM prediction that follows, such as skin type, treatment status, dominant hand, and race (Table 2).

**Table 2.** Baseline study questionnaire and test descriptions collected from the study’s participants.

Questionnaire and test	Description of content	References
Demographic	Five items: age, gender, ethnicity, handedness, an Fitzpatrick skin type	[13]
Diabetic status and history	Six items: family history, status, year diagnosed, medication, gestational diabetes, and drink consumption	[13,20-23]
Relevant medical history	Four items: smoking, hypercholesterolemia, hypertension, and related medication	[13,21-23]
Physical activity	International Physical Activity Questionnaire (IPAQ) long form; 27 items	[24]
Clinical measurements	Four items: weight, height, waist circumference, and blood pressure	[13,20,21,23]
Pre-photoplethysmography measurements questions	Seven items: time of medication, time of meal, meal content, physical activity in 24 h, last, duration, and intensity	[12,25]

### Ethical Considerations

All protocols were performed under hospital’s medical staff supervision at all times. Ethics approval for the study has been obtained from the Ukrida Faculty of Medicine & Health Sciences ethical committee on July 15, 2024 (1779/SLKE/IM/UKKW /FKIK/KEPK /VII/2024).

### Blood Sample Analysis

All capillary blood samples were analyzed using the Accu-Chek Guide glucometer (Roche, USA). Cubital plasma blood samples were analyzed for full blood count using the Sysmex XN-550 (Sysmex, Japan), plasma blood glucose using the Cobas C111 Analyzer (Roche, Switzerland), and HbA_1c_ levels using the FineCare FIA Meter III Plus (Gongu Wondfo, China). These instruments were available at the Ukrida Hospital clinical laboratory. All hospital equipment was routinely calibrated according to the provider’s manual.

### Photoplethysmography Data Collection

In this study, 2 wearable devices were utilized: the Actxa Spark+Series 2 smartwatch and the JC Smart Health Ring 2301B. Both devices are equipped with a green light photoplethysmography sensor that captures photoplethysmography signals at 50 Hz, ensuring adequate signal quality for HR analysis.

During data collection, the smartwatch was worn on the participant’s nondominant wrist, positioned at least 2 finger widths away from the wrist bone. The smart ring, selected based on appropriate sizing, was worn at the base of the index finger on the same hand. To ensure optimal signal acquisition, the ring’s photoplethysmography sensor was positioned facing the inner side of the finger. Each photoplethysmography recording session lasted for 5 minutes. Participants were instructed to remain seated, maintain a relaxed posture, and minimize movement, with their nondominant hand resting on a table to reduce motion artifacts.

Photoplethysmography data were collected by the wearable devices and transmitted via Bluetooth to a smartphone. The smartphone then relayed the data via the internet to Actxa’s company server for storage and further analysis.

### Non-Photoplethysmography Data Preprocessing

All 7 non-photoplethysmography features required for downstream model training—age, gender, body weight, body height, meal timing, diabetes status, and family history of diabetes—were complete and free of errors. Automated blood test results and finger prick glucometer measurements also contained no missing or invalid values.

Medication status, collected via participant self-report, was manually curated by medical personnel. Other questionnaire data will be further cleaned or curated as needed for downstream analyses. Missing values, if encountered, can be addressed by tracing the source information or applying statistical methods, such as decision tree–based imputation.

### Photoplethysmography Data Preprocessing

Each photoplethysmography recording was required to be at least 4 minutes in duration. No recordings from this clinical trial were excluded based on this criterion.

One participant was absent for the fourth measurement (3 h after the meal). For this participant, the available time point data were retained for individual data point analyses. However, in the time-series analysis (see the section photoplethysmography Data Feature Extraction), the data were excluded due to the incomplete sequence.

Raw photoplethysmography signals were obtained from the smartwatch (16-bit binary) and smart ring (23-bit binary) devices. Raw photoplethysmography data were preprocessed using a previously developed pipeline incorporating frequency noise filtering and outlier correction [12].

During the signal preprocessing stage, the raw digital signals were first converted to analog values using the following formula:

$V_{i}=5\;\times \frac{Signal}{2^{16}}$  (for smartwatch data)

$V_{i}=5\;\times \frac{Signal}{2^{23}}$  (for smart ring data)

A Chebyshev Type II bandpass filter (0.3‐5 Hz) was applied to remove low- and high-frequency noise, including motion artifacts. Outliers were identified, when necessary, using a *z* score threshold of 3 SD from the mean and replaced with reasonable estimates via nearest-neighbor interpolation prior to HR variability feature extraction.

### Photoplethysmography Data Feature Extraction

To accurately identify key temporal positions of the photoplethysmography waveform, potential systolic peaks were first detected using sign changes in the first-order difference of the photoplethysmography signal, specifically identifying transitions from positive to negative values. The leading and trailing troughs of the pulse wave were then detected by inverting the photoplethysmography signal and applying the same first-order difference and sign change method.

HR was then calculated using the key temporal positions. The measured HR was required to exceed 48 beats per minute. No recordings from this clinical trial were excluded based on this criterion.

Using the identified key temporal positions, a feature extraction module was then applied to the photoplethysmography data fragments to compute a comprehensive set of features. The panel of features was established in our previously reported model, which included 248 features related to HR, HR variability, waveform morphology, energy metrics, complexity measures, and continuous wavelet transform [12].

Prior to model training, feature selection techniques were applied to eliminate redundant features. This process was conducted exclusively on the feature set derived from photoplethysmography data and involved 2 stages. First, a self-correlation matrix was calculated, and features with a Pearson correlation coefficient >0.7 were removed to reduce multicollinearity. Second, the correlation between each remaining feature and the regression target—blood glucose levels measured via finger prick tests under different mealtime conditions—was assessed. A subset of features with the strongest correlations to the target variable was retained, with the final number determined empirically to optimize model performance.

To account for the varying feature magnitudes, Min-Max scaling was applied. Since the regression target (blood glucose values) exhibited a nonnormal distribution, a Box-Cox transformation was used for normalization. Following model prediction, an inverse Box-Cox transformation was performed to restore the predicted blood glucose levels to their original scale.

### Model Training

During model training, 10-fold cross-validation was applied to obtain a robust estimate of generalization performance and to monitor potential overfitting. To prevent data leakage, particularly biometric identity-related features potentially embedded in the photoplethysmography data [26], we ensured that fragments from the same participant were not included in both the training and validation sets. Data collected in this clinical trial were used exclusively for model training and validation.

Additionally, given that this study employs short-term, mealtime prospective measurements of blood glucose profiles, we aim to leverage each participant’s individual blood glucose fluctuation pattern to explore prediction of diabetes risk or glucose levels and probable glycemia monitoring post meal. To achieve this, the photoplethysmography data from the 4 mealtime points for each participant will be combined into a time series, which will serve as input to a model trained to predict glucose levels or diabetes risk across all 4 points of the meal cycle. This approach allows the longitudinal aspect of the data—the participant-specific fluctuation pattern—to be fully utilized, potentially improving model performance and predictive accuracy.

### Statistical Analysis

Demographic data for continuous variables will be presented as mean (SD), and categorical variables will be presented as count (percentage). Univariate analyses will be performed using *t* tests for independent groups for continuous variables and chi-square tests for categorical variables. Discriminations are assessed by plotting the receiver operating characteristic curve and calculating the area under the receiver operating characteristic curve (AUC) or *C* statistic. The accuracy of the prediction model will be analyzed using both Type I and Type II Parkes Error Grids. The sensitivity, specificity, and *F*_1_-score of the BGEM model will also be evaluated and presented to give a clear description of its strength and weakness.

The Parkes Error Grid is used to assess how clinically risky an inaccurate blood glucose reading might be. Each device reading is compared with a reference value and placed into 1 of 5 zones. Zone A means the reading is clinically accurate and would not change treatment. Zone B reflects small errors that might adjust a decision but have little or no impact on outcome. Zone C includes errors likely to affect clinical results. Zone D involves inaccuracies that could lead to clearly risky treatment decisions. Zone E represents dangerous errors that could cause severe or life-threatening consequences if acted upon [27].

The ML model will be trained using Lasso regression, cross-validation, and other ML techniques to find the optimal regularization parameter to ensure that the model generalizes to unseen data. The dataset is initially divided into a training set and a validation set. The training set is used for model development and hyperparameter tuning, while the validation set is reserved for a final, unbiased evaluation of the model’s performance. Analysis will ensure that the model does not overfit to the training data and is able to predict outcomes on new data with confidence. Once the optimal regularization parameter is identified through cross-validation, a final regression model is trained on the entire training set using this optimal regularization parameter value.

The blood sugar profiles of subjects with varying demographic characteristics will be analyzed and compared statistically to quantify the similarities and differences between individuals. Profile analysis and correlation and regression techniques will be used for the comparison of the blood sugar profiles, taking into consideration the correlation between the repeated measurements within each individual. Trending analysis will also be performed using mixed effects models and clustering methods, so as to incorporate the correlated repeated measurements at the 4 time points for each participant.

The data will be analyzed using statistical packages, including Stata (version 19) and ML packages R and Python. *P* values <.05 will be considered as statistically significant.

## Results

The IRB approval was given on July 15, 2024, after which the data collection was started on July 30, 2024, and finished on October 5, 2024; the analysis was projected to be concluded in the third quarter of 2025 and the results submitted for publication at the end of 2025.

A total of 885 participants (473 with diabetes and 412 without diabetes) were enrolled. Eight photoplethysmography recordings per participant were collected across 4 mealtime points using 2 wearable devices. Participants completed the International Physical Activity Questionnaire (IPAQ) long form and additional questionnaires on diabetes and related risk factors. Capillary and plasma blood tests were performed, and results were recorded.

## Discussion

### Overview

This study will provide more data to further empower the BGEM AI model that has been developed since 2022 [12].

Photoplethysmography feature data from participants, collected across 4 time points, are intended to support the improvement of model performance across diverse racial groups, risk factors, and skin types in terms of its accuracy and robustness. The study design also addresses issues that have been reported to influence photoplethysmography sensors, such as motion artifacts.

The inclusion of additional clinical data such as anthropometric measurement, HbA_1c_, hematocrit, and other blood parameters performed in this study will give a more expansive view on factors that may influence photoplethysmography blood glucose predictive ability [14-16,28]. Analysis of these factors will provide insight into whether any of them influence BGEM accuracy or can be used as additional data to improve its performance.

This study is the first to report performance of photoplethysmography-based AI model used in the Indonesian population. Population-wide DM screening in Indonesia posed a problem to any health intervention program due to the challenging geography and population spread across multiple islands and diverse ethnicity of Indonesian people [29,30]. Therefore, this study can become a foundation of BGEM use in DM management, prevention, and screening in Indonesia.

DM, with its huge disease burden and increasing number of afflicted populations, needs new, innovative, and effective means to control and minimize its impact [31,32]. This protocol paper hopes to provide a base method in developing a noninvasive photoplethysmography-based AI model to predict blood glucose level and evaluating DM status of the subject.

### Comparison With Previous Work

BGEM use in the relatively large Indonesian population is unique and will become an important stepping stone for developing a noninvasive model that is effective in blood glucose screening and monitoring and DM management. The previous study of BGEM was performed in a female-dominant and mainly non-DM population [13]; thus, this study with a different gender make-up and larger DM population (Table 3) will give a good contrast and data set with different population characteristics that may increase the model performance and generalizability [12,28].

**Table 3.** Comparison of basic population characteristics between the previous clinical study at KK Women’s Hospital, Singapore, and the current study in Krida Wacana Christian University (Ukrida) Hospital, Indonesia.

Characteristics	KK Women’s Hospital	Ukrida Hospital
Age (y), mean (SD)	38.73 (10.60)	44.22 (11.79)
Female proportion, n/N (%)	436/500 (90.3)	535/885 (60.45)
BMI (kg/m^2^), mean (SD)	24.4 (5.13)	26.77 (5.08)
Type 2 DM^a^ proportion, n/N (%)	157/500 (31.40)	473/885 (53.44)

a DM: diabetes mellitus.

The study has a comprehensive 4 time points of data collection, which is more extensive [33,34], and will provide a more complete view of how blood glucose level fluctuates between different state of food intake. The additional 1 and 3 hours after the meal may give a clearer view of blood glucose level changes compared to the standard fasting and 2-hour postmeal that is already widely accepted. Additionally, the use of AI in assessing glycemic variability is very limited [35], and to our knowledge, no study using photoplethysmography-based sensors has been reported despite its strong potential use for DM management. Therefore, this study will also be giving new and novel insight on the topic.

### Study Limitations

 This protocol has certain limitations. We prioritized achieving a balanced distribution of gender and participants with and without diabetes. With this focus, efforts were made to increase the overall number of participants. As the sample size grew, a broader range of demographic characteristics was naturally captured, reflecting the inherent diversity of the larger Indonesian population. However, no deliberate strategies were employed to design or randomize demographic features; the observed diversity emerged organically from the expanded participant pool. Several demographic features, such as participants aged over 60, are underrepresented; this will undermine the understanding of photoplethysmography in BGEM’s predictive ability in elderly population. Although the study includes a diverse range of ethnicities, the overall ethnic composition does not reflect the general proportions of the Indonesian population. This limits the extent to which certain inferences can be drawn from the final data analysis. Future studies with more proportional sampling from the general Indonesian population should address this bias.

### Conclusions

This protocol paper outlines the methodology designed for evaluating BGEM, a photoplethysmography-based AI model designed to estimate blood glucose levels and diabetic risk. The clinical trial was conducted on Indonesian participants with and without DM, while considering various influencing factors. The protocol is distinctive as it aims to generate high-quality training data for the AI model, ensuring balanced data structure, sufficient data quantity, diversity, and comprehensive relevant information including unique data on glycemic variability using wearables for downstream analysis and future research.

## Supplementary material

10.2196/76558 Multimedia Appendix 1 Nutritional value breakdown of each ingredient from the meal set given to break the subjects' fast.

## Abbreviations

AI: artificial intelligence
BGEM: Blood Glucose Evaluation and Monitoring
DM: diabetes mellitus
HbA_1c_: hemoglobin A_1c_
HR: heart rate
ML: machine learning
Ukrida: Krida Wacana Christian University
