# Supplementary material for: Assessment of Blood Glucose Measurement Using New Noninvasive Technology: Protocol and Methodology
Source: JMIR Res Protoc. 2026 Jan 8;15:e76558. doi: 10.2196/76558 (PMC12782458; doi:10.2196/76558)
Supplement: Multimedia Appendix 1 [file resprot-v15-e76558-s001.docx]

Multimedia Appendix

Table S1. Nutritional value breakdown of each ingredient from the meal set given to break the subjects fast

| **MEAL 1** | **INGREDIENTS** | **WEIGHT (g)** | **MEAL ANALYSIS** | | | | | | | | | | | | | | | | | | | | | | | | | | |
| --- | --- | --- | --- | --- | --- | --- | --- | --- | --- | --- | --- | --- | --- | --- | --- | --- | --- | --- | --- | --- | --- | --- | --- | --- | --- | --- | --- | --- | --- |
|  |  |  | **Energy (kcal)** | **Water (g)** | **Protein (g)** | **%** | **Fat (g)** | **%** | **Carb. (g)** | **%** | **Dietary fiber  (g)** | **PUFA  (g)** | **Cholesterol (mg)** | **Vit. A (µg)** | **Carotene (mg)** | **Vit. E (eq.) (mg)** | **Vit. B1 (mg)** | **Vit. B2 (mg)** | **Vit. B6 (mg)** | **Tot. fol.acid (µg)** | **Vit. C (mg)** | **Sodium (mg)** | **Potassium (mg)** | **Calcium (mg)** | **Magnesium (mg)** | **Phosphorus (mg)** | **Iron (mg)** | **Zinc (mg)** |  |
| Oriental fried rice with chicken, vegetable and chili sauce | Boiled rice | 150 | 195 | 0 | 3.6 | 8 | 0.3 | 1 | 42.9 | 89 | 0.5 | 0.2 | 0 | 0 | 0 | 0 | 0 | 0 | 0.1 | 3 | 0 | 0 | 43.5 | 4.5 | 19.5 | 55.5 | 0.3 | 0,6 |  |
|  | Chicken breast | 50 | 50.9 | 37.3 | 11.8 | 94 | 0.3 | 6 | 0 | 0 | 0 | 0.1 | 33 | 13.5 | 0 | 0.1 | 0 | 0 | 0.3 | 4.5 | 0 | 36 | 165 | 7 | 13.5 | 105 | 0.3 | 0,3 |  |
|  | Flour | 15 | 54.6 | 0 | 1.5 | 11 | 0.2 | 2 | 11.4 | 85 | 0.4 | 0.1 | 0 | 0 | 0 | 0 | 0 | 0 | 0 | 2.7 | 0 | 0.3 | 16 | 2.3 | 3.3 | 16.2 | 0.2 | 0,1 |  |
|  | Cooking oil 1 | 6 | 51.7 | 0 | 0 | 0 | 6 | 103 | 0 | 0 | 0 | 0.1 | 0 | 300 | 0 | 0.2 | 0 | 0 | 0 | 0 | 0 | 0 | 0 | 0.4 | 0 | 0.5 | 0 | 0 |  |
|  | Carrot | 5 | 1.3 | 4.5 | 0 | 15 | 0 | 7 | 0.2 | 76 | 0.2 | 0 | 0 | 78.7 | 0.4 | 0 | 0 | 0 | 0 | 0.6 | 0.3 | 3 | 14.5 | 2 | 0.9 | 1.8 | 0.1 | 0 |  |
|  | String beans | 5 | 1.7 | 0 | 0.1 | 22 | 0 | 8 | 0.4 | 92 | 0.2 | 0 | 0 | 3.3 | 0 | 0 | 0 | 0 | 0 | 1.6 | 0.5 | 0.2 | 14.9 | 2.3 | 1.3 | 2 | 0.1 | 0 |  |
|  | Corn kernel | 5 | 5.4 | 0 | 0.2 | 12 | 0.1 | 11 | 1.3 | 94 | 0.1 | 0 | 0 | 0 | 0 | 0 | 0 | 0 | 0 | 2.3 | 0.3 | 0.9 | 12.4 | 0.1 | 1.6 | 5.2 | 0 | 0 |  |
|  | Cooking oil 2 | 10 | 86.2 | 0 | 0 | 0 | 10 | 103 | 0 | 0 | 0 | 0.2 | 0 | 500 | 0 | 0.4 | 0 | 0 | 0 | 0 | 0 | 0 | 0 | 0.6 | 0 | 0.7 | 0 | 0 |  |
|  | Salt | 2.5 | 0 | 0 | 0 |  | 0 |  | 0 |  | 0 | 0 | 0 | 0 | 0 | 0 | 0 | 0 | 0 | 0 | 0 | 968.1 | 0.2 | 1.1 | 0.1 | 0 | 0 | 0 |  |
|  | Pepper | 2 | 6.5 | 0 | 0.3 | 16 | 0.3 | 38 | 1.2 | 73 | 0.7 | 0.1 | 0 | 2 | 0 | 0 | 0 | 0 | 0 | 5.6 | 0.2 | 1 | 30.9 | 9.6 | 5.1 | 7 | 0.6 | 0,1 |  |
|  | Chili | 12 | 38.2 | 0 | 1.4 | 15 | 2.1 | 48 | 6.8 | 72 | 3 | 1 | 0 | 499.3 | 0 | 0.1 | 0 | 0.1 | 0.2 | 10.6 | 9.1 | 3.6 | 241.7 | 17.8 | 18.2 | 35.2 | 0.9 | 0,3 |  |
|  | Garlic | 3 | 2.6 | 0 | 0.1 | 13 | 0 | 4 | 0.6 | 94 | 0.1 | 0 | 0 | 0 | 0 | 0 | 0 | 0 | 0 | 0.9 | 0.3 | 0.2 | 10 | 1.3 | 0.7 | 2.1 | 0 | 0 |  |
| Iced jasmine tea | Sugar | 15 | 58 | 0 | 0 | 0 | 0 | 0 | 15 | 105 | 0 | 0 | 0 | 0 | 0 | 0 | 0 | 0 | 0 | 0 | 0 | 0.2 | 0.3 | 0.2 | 0 | 0.3 | 0 | 0 |  |
|  | Drinking water | 200 | 0 | 200 | 0 |  | 0 |  | 0 |  | 0 | 0 | 0 | 0 | 0 | 0 | 0 | 0 | 0 | 0 | 0 | 2 | 0 | 10 | 2 | 0 | 0 | 0,2 |  |
| **Total** | | 480,5 | 552.1 | 241.8 | 19.0 | 206.0 | 19.3 | 331.0 | 79.8 | 780.0 | 5.2 | 1.8 | 33.0 | 1396.8 | 0.4 | 0.8 | 0.0 | 0.1 | 0.6 | 31.8 | 10.7 | 1015.5 | 549.4 | 59.2 | 66.2 | 231.5 | 2.5 | 1.6 |  |
